# YOLOv8-TF: Transformer-Enhanced YOLOv8 for Underwater Fish Species Recognition with Class Imbalance Handling

**DOI:** 10.3390/s25061846

**Published:** 2025-03-16

**Authors:** Chiranjibi Shah, M M Nabi, Simegnew Yihunie Alaba, Iffat Ara Ebu, Jack Prior, Matthew D. Campbell, Ryan Caillouet, Matthew D. Grossi, Timothy Rowell, Farron Wallace, John E. Ball, Robert Moorhead

**Affiliations:** 1 Northern Gulf Institute, Mississippi State University, Starkville, MS 39759, USA; jhp277@ngi.msstate.edu (J.P.); rjm@gri.msstate.edu (R.M.); 2 The School of Engineering and Applied Sciences, Western Kentucky University, Bowling Green, KY 42101, USA; mm.nabi@wku.edu; 3 Department of Electrical and Computer Engineering, James Worth Bagley College of Engineering, Mississippi State University, Starkville, MS 39762, USA; sa1724@msstate.edu (S.Y.A.); ie93@msstate.edu (I.A.E.); 4 National Marine Fisheries Services, Southeast Fisheries Science Center, 3209 Frederic Street, Pascagoula, MS 39567, USA; matthew.d.campbell@noaa.gov; 5 NOAA Fisheries, 4700 Avenue U, Galveston, TX 77551, USA; ryan.caillouet@noaa.gov (R.C.); matt.grossi@noaa.gov (M.D.G.); timothy.rowell@noaa.gov (T.R.); farron.wallace@noaa.gov (F.W.)

**Keywords:** fish species recognition, SEAMAPD21, YOLOv8-TF, object detection

## Abstract

In video-based fish surveys, species recognition plays a vital role in stock assessments, ecosystem analysis, production management, and protection of endangered species. However, implementing fish species detection algorithms in underwater environments presents significant challenges due to factors such as varying lighting conditions, water turbidity, and the diverse appearances of fish species. In this work, a transformer-enhanced YOLOv8 (YOLOv8-TF) is proposed for underwater fish species recognition. The YOLOv8-TF enhances the performance of YOLOv8 by adjusting depth scales, incorporating a transformer block into the backbone and neck, and introducing a class-aware loss function to address class imbalance in the dataset. The class-aware loss considers the count of instances within each species and assigns a higher weight to species with fewer instances. This approach enables fish species recognition through object detection, encompassing the classification of each fish species and localization to estimate their position and size within an image. Experiments were conducted using the 2021 Southeast Area Monitoring and Assessment Program (SEAMAPD21) dataset, a detailed and extensive reef fish dataset from the Gulf of Mexico. The experimental results on SEAMAPD21 demonstrate that the YOLOv8-TF model, with a mean Average Precision (mAP)_0.5_ of 87.9% and mAP_0.5–0.95_ of 61.2%, achieves better detection results for underwater fish species recognition compared to state-of-the-art YOLO models. Additionally, experimental results on the publicly available datasets, such as Pascal VOC and MS COCO datasets demonstrate that the model outperforms existing approaches.

## 1. Introduction

Accurate fish species identification holds significant importance in fisheries management and environmental monitoring. It plays a vital role in various aspects, including the identification of endangered species, optimizing harvesting timing and size, ecosystem monitoring, and establishing efficient production management systems [1,2,3]. Accurate fish species recognition becomes even more crucial due to legal constraints on fishing methods, particularly for threatened or endangered species. Traditional approaches for identifying fish species often rely on manual, labor-intensive processes that consume substantial time. Furthermore, hook-based sampling can disturb the natural behavior of fish. Conventional methods present difficulties in ensuring the availability of reliable data to effectively manage sustainability in fisheries, monitoring federal fisheries, evaluating fish populations, and distinguishing between various species of fish. In contrast, leveraging computer vision, deep learning (DL) technology, and annotation tool offers the potential for robust models in the identification of fish species, leading to cost and time savings while enhancing the accuracy of identification [4,5,6,7].

Machine vision solutions have the potential to replace manual counting and species identification by offering comparable or even superior precision. Several methods for fish detection, including lidar [8,9], sonar [10], and RGB imaging [11], are available. When it comes to species identification in clear water, RGB imaging stands out as the favored choice. It enables a straightforward identification of fish using their color, texture, and geometry. Additionally, RGB imaging is not only cost-effective and lightweight, but is also environmentally friendly as it does not disrupt fish habitat. In earlier studies, the analysis of video frames [12] was performed on an individual basis to detect objects present in each frame. Recently, multiple camera systems have been employed to monitor fish stocks and assess the sustainability of marine ecosystems. Nevertheless, these systems face a common challenge of time-consuming manual processing bottlenecks [13]. Recent advancements in deep learning have demonstrated significant potential for extracting valuable information about marine ecology through object detection and classification [14,15,16]. Though RGB imaging offers advantages in clear water conditions, its effectiveness is significantly hindered by the challenges posed by underwater environments, such as low light and turbid conditions [17,18], occlusion [19], low-resolution images and videos, and the inherent difficulty in distinguishing fish from the background [20]. These factors can lead to blurry images and noisy data, making it difficult to accurately detect and classify objects, particularly smaller fish species. To address these issues, innovative approaches such as the SWIPENET+CMA framework have been proposed in [21]. By leveraging advanced DL techniques, this framework aims to improve the accuracy of object detection and classification in challenging underwater conditions. While optical cameras struggle in underwater environments characterized by poor lighting and turbidity, sonar cameras [22] offer a more reliable solution. Sonar systems can cover a wider area and penetrate deeper into the water column, making them suitable for large-scale underwater surveys. The integration of machine learning (ML) techniques with sonar data [23] has enabled the development of automated systems for analyzing sonar data, facilitating precise fish detection and classification [24]. However, the high cost of high-resolution 3D sonar cameras remains a significant barrier to their widespread adoption. The dynamic movement of fish introduces complexities such as shape variations and object overlaps, which pose challenges in accurately identifying and detecting underwater fish species. Another significant challenge for marine applications is marine snow, comprised of drifting organic and inorganic particles, significantly degrades underwater image clarity. Solutions targeting this issue include: end-to-end dual-channel frameworks that address multiple degradations including marine snow removal [25], deep learning approaches fusing spatial and Fourier domain information for effective marine snow restoration [26], and the introduction of a dedicated benchmarking dataset to facilitate further research in this area [27]. In addition, DL is a prominent field within computer vision, has found widespread application in addressing diverse challenges such as detection, localization, estimation, and classification [4,28,29,30,31]. Numerous ML and DL algorithms have been created for the purpose of categorizing fish species. For example, Jager et al. [32] utilized the AlexNet architecture for feature extraction and employed multiclass support vector machine (SVM) for classification. Similarly, hierarchical features combined with SVM have been utilized for fish classification. Carion et al. [33] introduced end to end object detection with transformers (DETR) and the experiments are conducted in challenging MSCOCO object detection datasets. Fang et al. [34] presented You Only Look Once (YOLO) model based on transformer for object detection on public MSCOCO dataset.

The You Only Look Once (YOLO) model [35,36,37,38] widely used for object detection, is effective in identifying fish, especially in videos. YOLO operates as a single-shot detection model [39], processing the entire image or video frame in one go to accurately predict object locations and classes. The YOLO model is specifically designed for efficient processing, making it ideal for real-time use. With extensive training on large datasets, it can achieve impressive levels of accuracy. The YOLO model utilizes a convolutional neural network (CNN) architecture and is trained using a set of labeled images. Through training, the model becomes adept at predicting bounding boxes that represent the location of fish in an image or video, along with the associated class probabilities (i.e., the fish species). Several loss functions are employed during training to penalize inaccurate predictions and encourage continuous improvement in the model’s prediction capabilities over time.

YOLO provides a unified framework for object detection, allowing it to identify multiple objects in one traversal of the network. This is advantageous for tasks that require detecting and recognizing multiple objects. YOLO offers the advantage of efficient processing by being highly parallelizable, allowing it to effectively utilize multiple GPUs simultaneously. However, due to its single-stage detection network architecture, YOLO sacrifices some precision compared to two-stage networks like Faster R-CNN [40]. Underwater images often contain numerous small objects, which are typically overlooked by the pooling layers in CNNs due to their size, making detection and recognition difficult. Moreover, similar to other object detection models, YOLO encounters challenges in accurately detecting fish in situations of low lighting or partial obstructions. Despite these limitations, YOLO continues to be widely favored and proven effective for tasks involving fish classification and detection [41]. The YOLOv5 technique for object detection was initially introduced by Jocher et al., 2021 [42], who utilized it on the MS COCO public datasets [43]. Subsequently, Jung et al., 2022 [44] implemented YOLOv5 specifically for object detection in drone images. Wang et al. [45] presented an optimized nighttime nail detection with an advanced YOLOv5 model for improved road safety. In our previous work [5], we enhanced YOLOv5 by modifying its backbone to improve fish species recognition.In addition, Varghese et al., 2024 [46] introduced a novel YOLOv8 model for object detection in the public MS COCO dataset. When compared to the aforementioned algorithms, YOLOv8 was a significant advancement in the YOLO series, known for its high accuracy, fast processing speed, and capacity to detect multiple objects within an image [47]. Following that, Li et al. [48] introduced a modified YOLOv8-based technique for recognizing objects in images collected from the air. Ansah et al. [49] proposed a SB-YOLO-V8 technique layered with deep Learning framework for real-time detection of humans. Bi et al. [50] introduced a streamlined detection network based on an improved YOLOv8 model, tailored for identifying small targets in drone-captured aerial images. The modifications aim to enhance detection accuracy for objects observed from high altitudes. None of the aforementioned techniques have been adequately implemented for recognizing fish species in underwater environments with highly imbalanced species distributions. In this study, we have proposed YOLOv8-TF: Transformer-Enhanced YOLOv8 for Underwater Fish Species Recognition with Class Imbalance Handling to better handle the challenges posed by class imbalance in underwater fish detection, further improving its effectiveness in these complex environments.

The primary contributions of this research are as follows:

1. To enhance fish species recognition, we made modifications to the depth scale of various layers within the backbone of the YOLOv8 model. These modifications resulted in improved performance and accuracy in identifying fish species.
2. We incorporated the transformer block into both the backbone and neck networks of the YOLOv8-based approach. This enhancement boosts the model’s capabilities by enabling it to capture more contextual and global information, thereby improving its performance.
3. To tackle the challenges posed by a highly imbalanced dataset, we introduced a class-aware loss function along with Wise-IoUv3 [48]. This approach enhances both classification and localization accuracy, leading to more precise and reliable results.

## 2. Related Works

The rapid rise in popularity of DL techniques has led to their successful implementation across various industries, including the fishery industry [51]. DL techniques have greatly influenced information and data processing in the realm of smart fish farming, presenting both new opportunities and challenges. Within aquaculture, DL is extensively utilized for various tasks, including live fish identification [52], species classification [53], behavioral analysis [54], feeding selection [55], size or biomass estimation [56], and water quality forecasting [57]. Several DL approaches exist that are particularly well-suited for fish datasets, allowing for the development of tailored DL models specific to each task.

The YOLO algorithm has gained significant popularity in fish identification and detection tasks. A notable early study conducted by Xu et al. (2018) [29] employed YOLO to develop a model capable of identifying fish in underwater videos. The study utilized three diverse datasets captured at water power sites to train the YOLO model specifically for this purpose. In a recent study, the Southeast Area Monitoring and Assessment Program Dataset (SEAMAPD21) was utilized to examine the effectiveness of YOLOv4 in the identification and categorization of different fish species. The results showcased the successful localization and identification of various fish species within the SEAMAPD21 dataset [58] using the YOLOv4 model. Alaba et al. [4] employed two feature extraction networks, namely MobileNetv3-large [59] and VGG16 [60], to extract relevant features from underwater images. They then utilized a single-shot multi-box detector (SSD) to classify fish species and detect the precise location of the fish within the images from the SEAMAPD21 dataset. Wang et al. [61] introduced a novel attention mechanism, Channel and Spatial Fusion Attention (CSFA), which integrates channel and spatial attention into the YOLOv5 architecture. By combining these attention mechanisms, the network can effectively focus on both the salient features and spatial context of detected objects, leading to improved detection performance. To overcome challenges like turbidity and low lighting in underwater object detection, Pachaiyappan et al. [62] worked on including attention mechanisms, transformers, and diffusion models. These approaches enhance image quality, enabling more accurate detection and classification by using advanced imaging technique YOLO network (AIT-YOLOv7), supporting Sustainable Development Goal (SDG) 14: Life Below Water, which aims to conserve and sustainably use the oceans, seas, and marine resources.

Sung et al. [63] introduced a real-time fish detection model based on the YOLO algorithm, specifically designed for underwater vision. The effectiveness and precision of the proposed method were evaluated using real fish video images. The CNN model achieved an impressive classification accuracy of 93%, a predicted bounding box intersection over a union of 0.634, and a fish detection rate of 16.7 frames per second. Notably, it outperformed a fish detector that utilized a sliding window algorithm and a classifier trained with a histogram of oriented gradient features and a SVM. Jalal et al. [64] presented a two-step DL approach for identifying and classifying temperate fishes. In the initial step, they employed the YOLO object detection method to detect each fish in the image, disregarding its species or gender. In the subsequent step, a CNN with the Squeeze-and-Excitation structure was utilized to detect each individual fish in the image without any preliminary filtering. To address the limited samples issue, transfer learning was employed to improve the overall classification accuracy.

Collectively, these studies highlight the potential of DL, specifically the YOLO-based approach, in fish identification and detection tasks. They showcase promising outcomes in terms of accuracy and processing speed. However, challenges remain in addressing issues related to the complex and diverse underwater environment, as well as improving the models’ ability to generalize and handle new and unseen fish species effectively.

## 3. Proposed Approach

### 3.1. A YOLOv8 Technique for the Recognition of Fish Species in Underwater Environments

**Figure 1:**
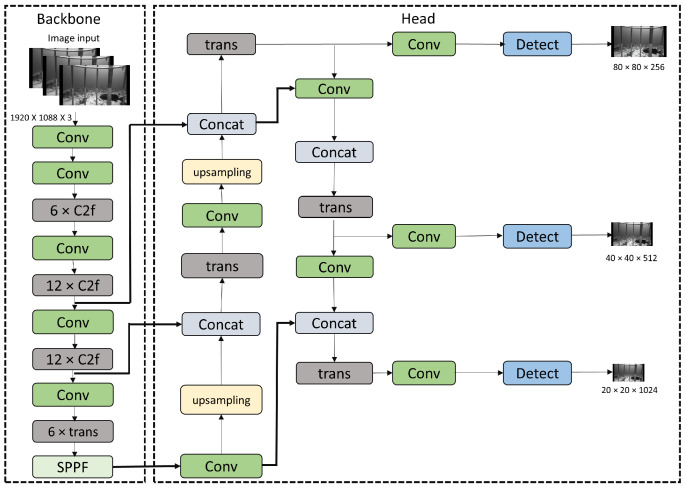
An enhanced architecture based on YOLOv8-TF for the detection of fish species using the SEAMAPD21 dataset.

**Figure 2:**
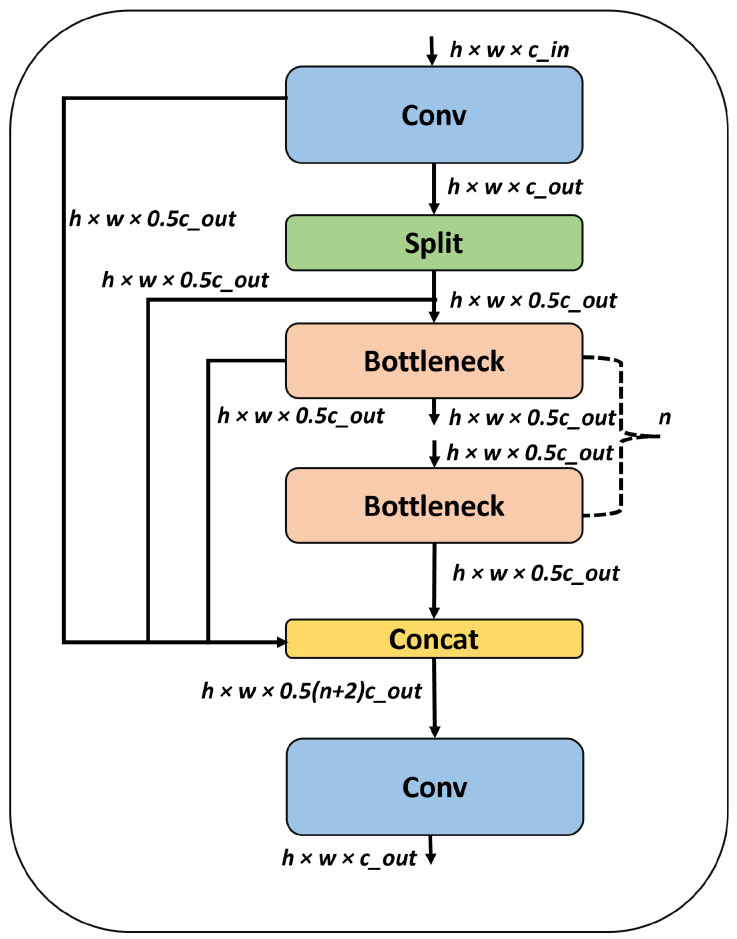
C2f block [68] for YOLOv8 model. For input image X with dimensions $h\times w\times c_{-}in$, the output of the C2f layer can be represented as Y with dimensions $h\times w\times c_{-}out$. It consists of n number of bottlenecks connected serially to transfer the information.

In YOLOv8, both the conventional convolution module and the C2f module [48,65] were employed to accomplish effective feature extraction and image downsampling, resulting in high-quality outputs, as shown in Figure 1. The C2f module integrates high-level features with contextual data to enhance detection performance. To gain richer gradient flow information while maintaining low weight, this module expands the gradient branch by reducing one conventional convolutional layer and fully utilizing the bottleneck module. YOLOv8 offers a range of models, including YOLOv8n, YOLOv8s, YOLOv8m, and YOLOv8l, each with varying architectural complexities and performance characteristics. While the underlying principle remains consistent, the classification of these models is based on their respective memory usage. For YOLOv8l, its backbone consists of C2f modules and conventional convolutional modules represented as 3×C2f−6×C2f−6×C2f−3×C2f. Figure 2 shows the detailed diagram for C2f module. Within the YOLOv8 architecture, feature maps are segregated into several distinct scale features in descending order. These features are denoted as C2f layers in the backbone, the FPN (Feature Pyramid Network) [66] structure and the PAN (Path Aggregation Network) [67] structure in the neck. The PAN-FPN structure employed in the YOLOv8 model serves as a complement to the conventional FPN. It utilizes a top-down approach to effectively transfer deep semantic features. In the final stage, the head component utilizes the image features obtained from the neck module to generate predictions, involving steps for both class and box predictions.

### 3.2. YOLOv8-TF: Transformer-Enhanced YOLOv8 for Underwater Fish Species Recognition with Class Imbalance Handling

The YOLOv8 backbone consists of C2f layers [48,65]. The C2f module establishes connections across layers through additional branches and adjusts varying channel numbers for different scale models. This design not only ensures a lightweight model but also captures richer gradient flow information. For the improvement in the backbone, we have modified the width and depth scales of the C2f layers. As shown in Figure 1, C2f modules for YOLOv8-TF model are represented as 6×C2f−12×C2f−12×C2f−6×C2f.

#### 3.2.1. Transformer Block

**Figure 3:**
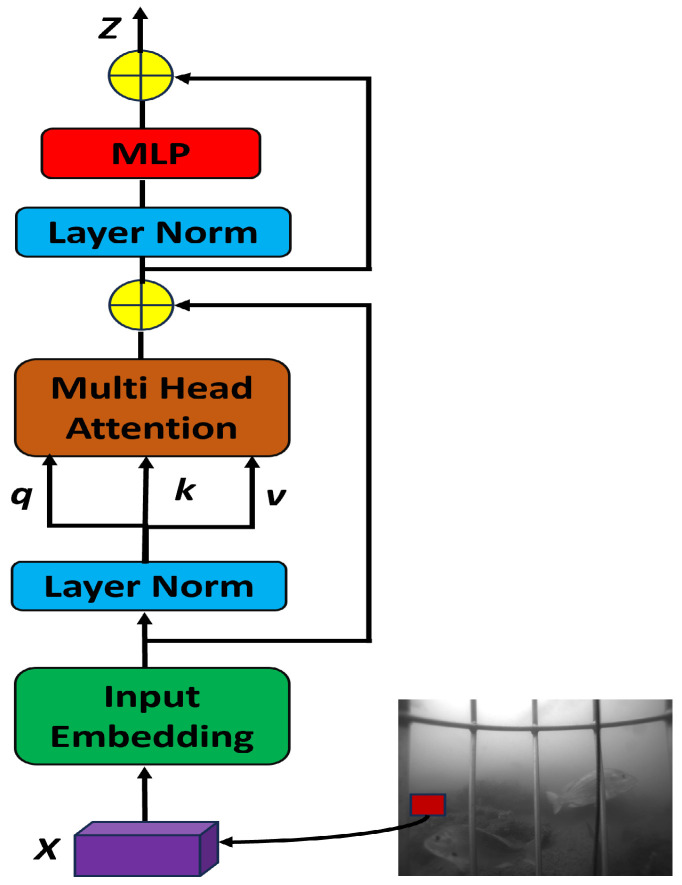
Transformer (trans) block for a YOLOv8-TF approach, where *q*, *k*, and *v* denote query, key, and value respectively. Here, ⊕ signify element-wise summation.

We have introduced a transformer (trans) block [5,69,70], as shown in Figure 3, in the backbone network of YOLOv8-TF resulting in C2f and transformer (trans) modules as 6×C2f−12×C2f−12×C2f−6×trans. For the improvements in the Neck, C2f modules are replaced with transformer (trans) modules. The transformer layers help capture long-range dependencies and global contextual information, which enhances the model’s ability to detect fish species more accurately by effectively distinguishing subtle visual patterns across different scales. For the processing of 2D images in trans layer, we flatten the spatial dimensions of the 2D feature map $x\epsilon R^{h\times w\times d}$ into a sequence $x_{p}\epsilon R^{h\times w\times d}$, where (h,w) represents the original feature map resolution, d denotes the number of channels, and h×w functions as the effective input sequence length for the transformer layer. To imbue the attention operation with positional awareness, transformer-based architectures commonly employ position encoding. We employ standard learnable 1D positional embeddings through a linear layer to maintain positional information.

#### 3.2.2. Wise-IoU Loss Function

YOLOv8 employs the anchor-free concept such that the Binary Cross Entropy (BCE) loss is used for the classification loss, and Bounding Box Regression (BBR) loss and distribution focal (DF) loss are used for the regression portion [48,65].

(1) $$L_{loss}=\alpha_{1}L_{BC}+\alpha_{2}L_{DF}+\alpha_{3}L_{BBR}$$

where $L_{BC}$ denotes classification loss, $L_{BBR}$ represents regression loss, and $L_{DF}$ defines the distribution focal loss with regularization coefficients $\alpha_{1}$, $\alpha_{2}$, and $\alpha_{3}$.

Wise Intersection over Union (WIoU) [71,72] introduces a dynamic, non-monotonic focus mechanism that evaluates anchor box quality through dissimilarity rather than traditional IoU. Then, the cost function can be estimated as:

(2) $$L_{loss}=\alpha_{1}L_{BC}+\alpha_{2}L_{DF}+L_{WIoUv3}$$

where $L_{WIoUv3}$ denotes the wise-IoU loss term, which can be estimated as below:

(3) $$L_{WIoUv3}=\left(1-\frac{\mathrm{bbox}\cap \mathrm{wbox}}{\mathrm{bbox}\cup \mathrm{wbox}}\right)\exp\;\left(\frac{{\left(x_{\mathrm{bbox}}-x_{\mathrm{wbox}}\right)}^{2}+{\left(y_{\mathrm{bbox}}-y_{\mathrm{wbox}}\right)}^{2}}{{\left(w^{2}+h^{2}\right)}^{★}}\right)\kappa$$

(4) $$\kappa =\theta /\tau \beta^{\theta -\tau }$$

The $L_{WIoUv3}$ loss evaluates the quality of an anchor box by considering both the spatial overlap and the distance between the centers of the predicted and ground truth boxes. Unlike traditional Intersection over Union (IoU) metrics, which only measure overlap, *wise IoU* introduces a distance-based penalty for more insightful gradient gain allocation. In this formulation, $x_{bbox},y_{bbox}$ represent the center coordinates of the predicted anchor box, while $x_{wbox},y_{wbox}$ denote the center coordinates of the ground truth anchor box. The terms bbox and wbox correspond to the predicted and ground truth bounding boxes, respectively, with *w* and *h* representing the width and height of the reference prediction box. The term θ represents the abnormality degree for prediction box, β and θ are hyperparameters. By incorporating the center distance into the loss calculation, *wise IoU* dynamically adjusts gradients to prioritize anchor boxes closer to the ground truth, ultimately improving object detection performance.

#### 3.2.3. Class-Aware Loss Function

To improve the loss function, we have introduced class-aware (ca) regularization [4] terms in classification, regression, and distribution focal loss terms of the YOLOv8 model.

(5) $$L_{loss}=\alpha_{1}L_{BCca}+\alpha_{2}L_{DFca}+L_{WIoUv3ca}$$

The proposed class aware classification, distribution focus, and regression loss terms can be defined as:

(6) $$\begin{array}{cc} L_{BCca} & =\frac{1-\left(\frac{n_{s}}{n}\right)}{1-{\left(\frac{n_{s}}{n}\right)}^{\eta }}L_{BC},\\ L_{DFca} & =\frac{1-\left(\frac{n_{s}}{n}\right)}{1-{\left(\frac{n_{s}}{n}\right)}^{\eta }}L_{DF},\\ L_{WIoUv3ca} & =\frac{1-\left(\frac{n_{s}}{n}\right)}{1-{\left(\frac{n_{s}}{n}\right)}^{\eta }}L_{WIoUv3}, \end{array}$$

where, $L_{BCca}$ is class-aware classification loss and $L_{WIoUv3ca}$ is class-aware wise IoU loss, and $L_{DFca}$ is the class-aware distribution focal loss. The quantity $n_{s}$ represents the number of training instances associated with each species, while *n* denotes the total number of training samples and η is a tunable hyper-parameter described below. It is important to note that in this context, $n_{s}$ is considerably smaller than *n*.

When $n_{s}$ is characterized by a small value, and the parameter η is increased, the multiplying term’s value $\left(\frac{1-\left(\frac{n_{s}}{n}\right)}{1-{\left(\frac{n_{s}}{n}\right)}^{\eta }}\right)$ increases as well. This increase effectively amplifies the weight assigned to the less-represented class. To achieve optimal performance, it is essential to fine-tune the hyper-parameter values. In most cases, raising the η value results in an increase in the value of the multiplying term within the loss functions. However, this alone may not lead to an overall performance improvement, as it can have repercussions on other classes. Consequently, the ideal hyper-parameter value for maximizing performance on a specific dataset must be systematically adjusted, beginning with an initial setting of parameter η = 1. For this specific training scenario, we set η to the value of 8.

### 3.3. Dataset Description

**Figure 4:**
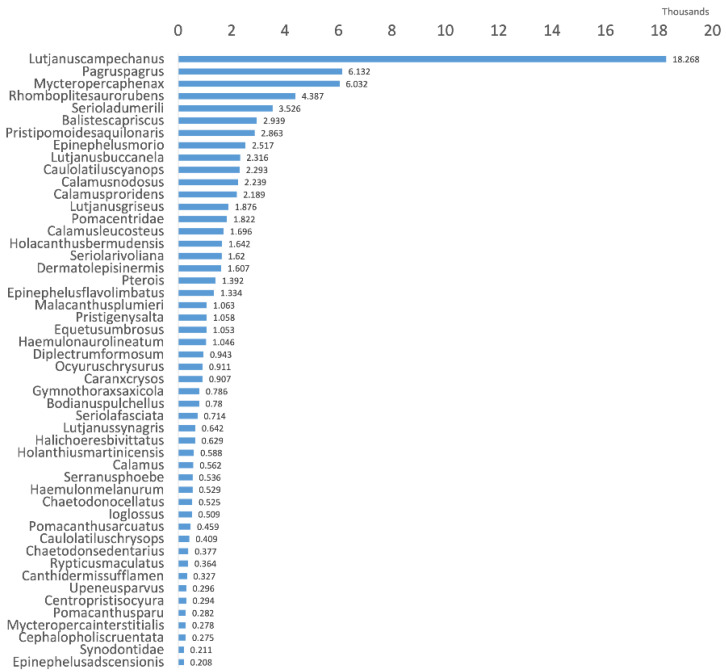
The distribution of sample occurrences per species in SEAMAPD21 [58] exhibits a highly imbalanced structure.

**Figure 5:**
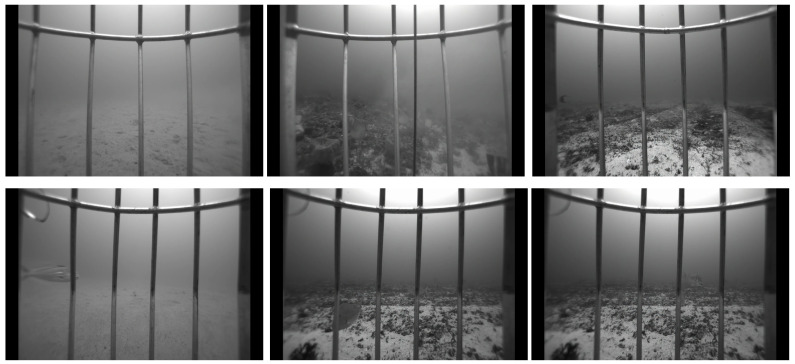

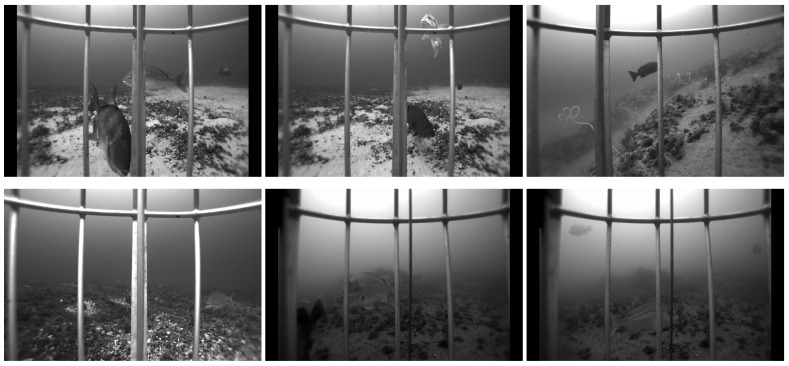
Images from the SEAMAPD21 dataset depict scenes where fish are often difficult to distinguish from the background, adding to the challenge of identification. Some fish images pose challenges even for human detection, possibly due to occlusion by vertical bars or other fish.

The Southeast Area Monitoring and Assessment Program Dataset 2021 (SEAMAPD21) [58] provides a large-scale collection of reef fish data from the Gulf of Mexico. This dataset encompasses 130 distinct classes of fish species captured in underwater environments, with a total of 28,319 images. However, certain species have limited representation in the dataset as shown in Figure 4, and the model’s performance is influenced by samples with a higher number of species per class. Furthermore, detecting fish in low-resolution underwater environments presents challenges due to the difficulty in distinguishing between images and the background. Images from the dataset are illustrated in Figure 5. The task of detecting fish in certain images presents challenges, even for a human. For the experimental setup, a train-validation-test split ratio of 70/15/15 is utilized. The mean average precision (mAP) is then evaluated on the test set to assess the model’s performance.

## 4. Experimental Results

For the methods in comparison, parameters were tuned according to MobileNetv3-large [4], VGG300 [4], VGG512 [4], YOLOV5s [5], YOLOv5m [5], YOLOV5l [5], YOLOV8s [65], YOLOV5enh [5], YOLOv8m [65], and YOLOV8l [65].

### 4.1. Implementation Details

For training the proposed YOLOv8-TF, we utilized PyTorch 1.13.0 and employed an NVIDIA A100-SXM GPU. The models were trained and tested using this setup. The approach was trained from scratch, and Stochastic Gradient Descent (SGD) was employed as the optimizer for the model. A total of 300 epochs were conducted during the training process. The values for η were varied from 1 to 16 and optimal performance was found at η = 8. Optimal values of $\alpha_{1}$ = 0.5, $\alpha_{2}$ = 1.5, $\alpha_{3}$ = 7.5, θ = 2, β = 1.8, and τ = 3 were used.

### 4.2. Performance

To evaluate the performance of the proposed YOLOv8-TF and other existing versions of YOLOv8, the commonly used metric mean average precision (mAP) is employed. Two variants of mAP are utilized: mAP_0.5:0.95_, which calculates the average precision over a range of Intersection Over Union (IoU) values from 0.5 to 0.95, and mAP_0.5_, which focuses on IoU at 0.5. These metrics provide a comprehensive assessment of the detection performance across different IoU thresholds. Table 1 presents the mAP for the YOLOv8enh-based approach in contrast to alternative methods. Certain species exhibit a limited number of samples, such as fewer than 10, which may result in inadequate samples for training, validation, and testing sets. As a result, 121 species are employed to gauge the mAP. The results indicate that the proposed YOLOv8-TF surpasses other versions, including YOLOv8n, YOLOv8s, YOLOv8m, YOLOv8l, YOLOv5s, YOLOv5m, YOLOv5l, MobileNetv3-large, VGG300, and VGG512 in the detection of fish species in underwater environments. Specifically, YOLOv8-TF exhibits superior performance with a mAP_0.5_ of 87.9% and mAP_0.5:0.95_ of 61.2%.

Additionally, Table 1 displays the network size, measured in terms of the number of parameters in millions (M), the number of calculations in GFLOPS, and speed measured in terms of Frames Per Second (FPS). It displays the inference latency on an NVIDIA A100-SXM GPU. This facilitates a comparison of the impact of the proposed approach with existing YOLOv8-based techniques. It is noticeable that YOLOv8-TF exhibits a higher number of parameters (51.08 M) and GFLOPS (165.4) compared to YOLOv8n, YOLOv8s, YOLOv8m, YOLOv8l, YOLOv5s, YOLOv5m, and YOLOv5l. YOLOv8 outperforms YOLOv5 in terms of speed measured with FPS for fish species recognition in underwater environments. YOLOv8-TF provides a competitive FPS of 116 compared to other methods. When considering accuracy measured in terms of mAP, YOLOv8-TF also demonstrates superiority compared to other methods.

**Figure 6:**
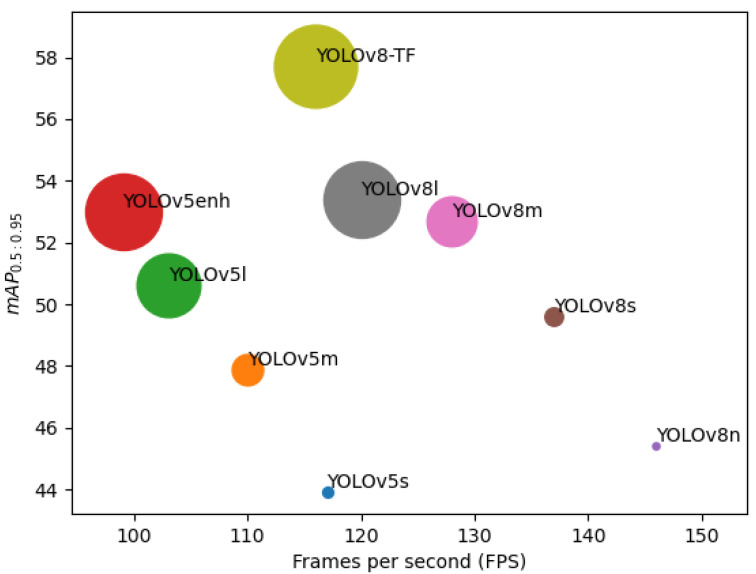
Comparing the performance of our proposed YOLOv8-TF with YOLOv5 and YOLOv8-based approaches for the SEAMAPD21 data based on FPS. GFLOPS is shown by radii of the circles.

Figure 6 presents a comparison between YOLOv8-TF and other existing versions like YOLOv5n, YOLOv5s, YOLOv5m, YOLOv5l, YOLOv5enh, YOLOv8s, YOLOv8m, and YOLOv8l focusing on metrics such as mAP, frames per second (FPS), and GFLOPS. The GFLOPS is depicted through the radii of the circles. While YOLOv8-TF demands higher GFLOPS compared to YOLOv8m, the performance improvement, measured in terms of mAP, makes up for this difference. In terms of speed, YOLOv8-TF with 116 FPS outperforms YOLOv5m, YOLOv5l, YOLOv5enh although it is slower compared to YOLOv8n, YOLOv8s, YOLOv8m, and YOLOv8l. The trade-off between detection accuracy and speed provided by YOLOv8-TF makes it superior in many cases compared to other versions.

Table 2 shows that the proposed YOLOv8-TF with a mAP_0.5_ of 94.30% and mAP_0.5:0.95_ of 58.10% outperforms all other compared methods for the publicly available Pascal VOC dataset. Additionally, parameters (in millions) and speed measured in FPS (frames per second) are reported. Proposed YOLOv8-TF shows better accuracy compared to other methods in expense of higher parameters 30.51M and lower FPS of 130. Similarly, Table 3 shows that the proposed YOLOv8-TF with a mAP_0.5_ of 69.50% and mAP_0.5:0.95_ of 51.80% outperforms all other compared methods for the publicly available MSCOCO dataset. Moreover, proposed YOLOV8-TF outperforms other compared methods with high mean average precision although it introduced complexity with higher parameters of 30.54M and slower FPS of 139.

**Figure 7:**
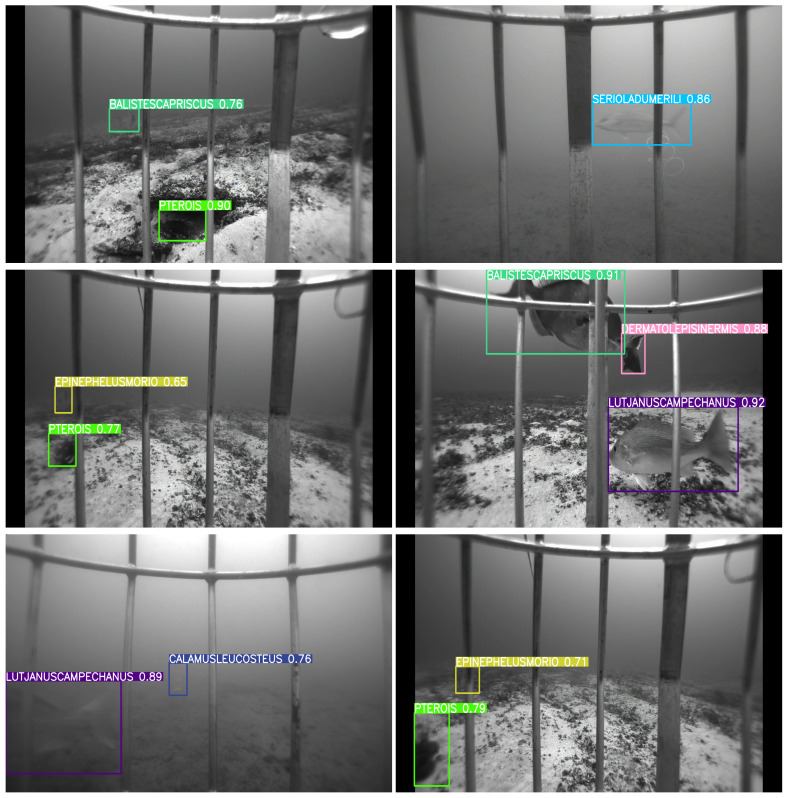
Detection images for YOLOv8s in SEAMAPD21.

**Figure 8:**
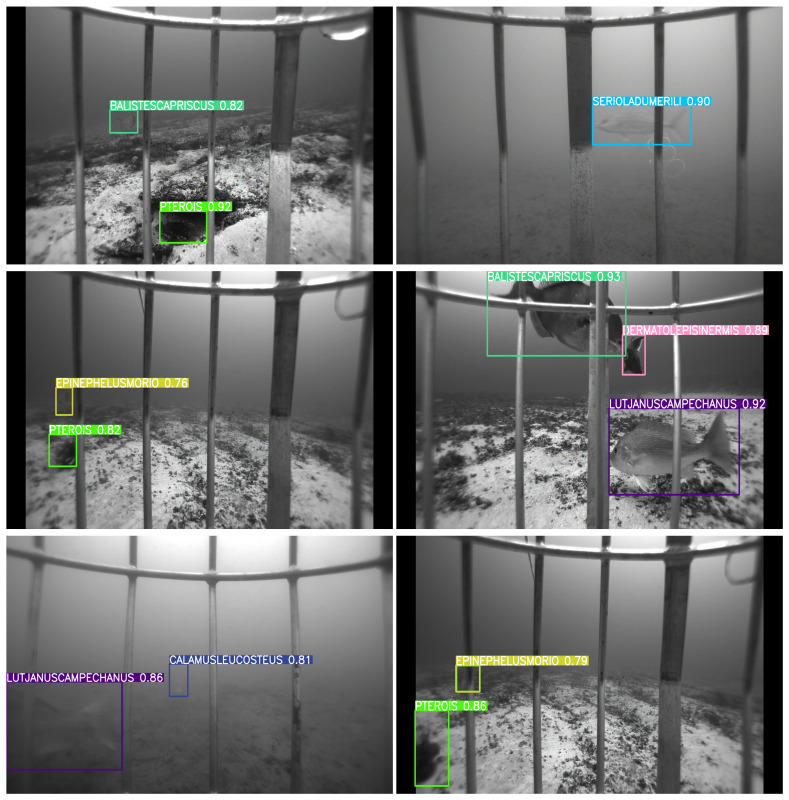
Detection images for YOLOv8m in SEAMAPD21.

**Figure 9:**
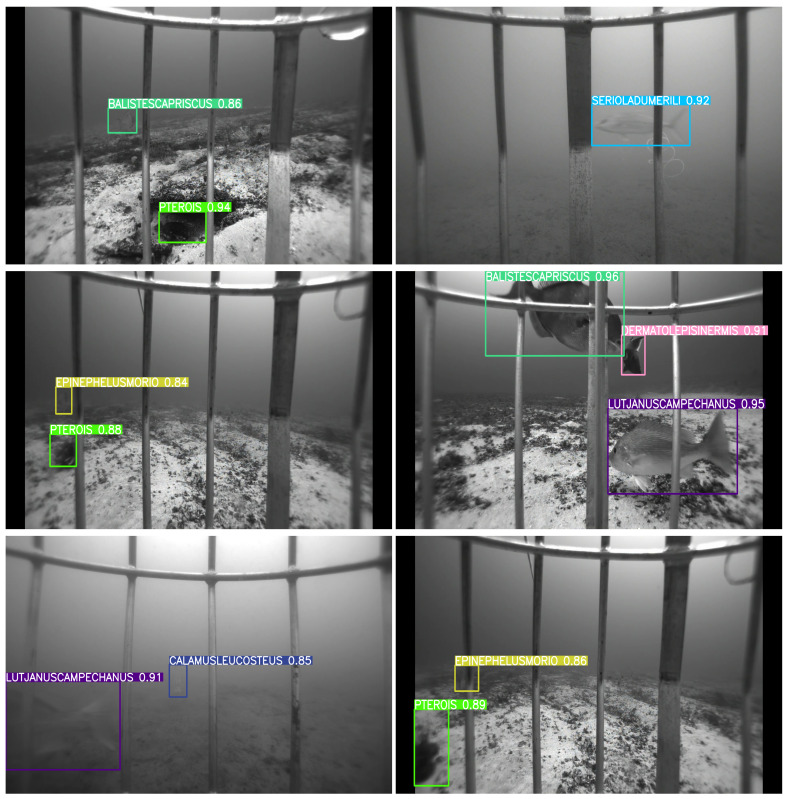
Detection images for YOLOv8l in SEAMAPD21.

**Figure 10:**
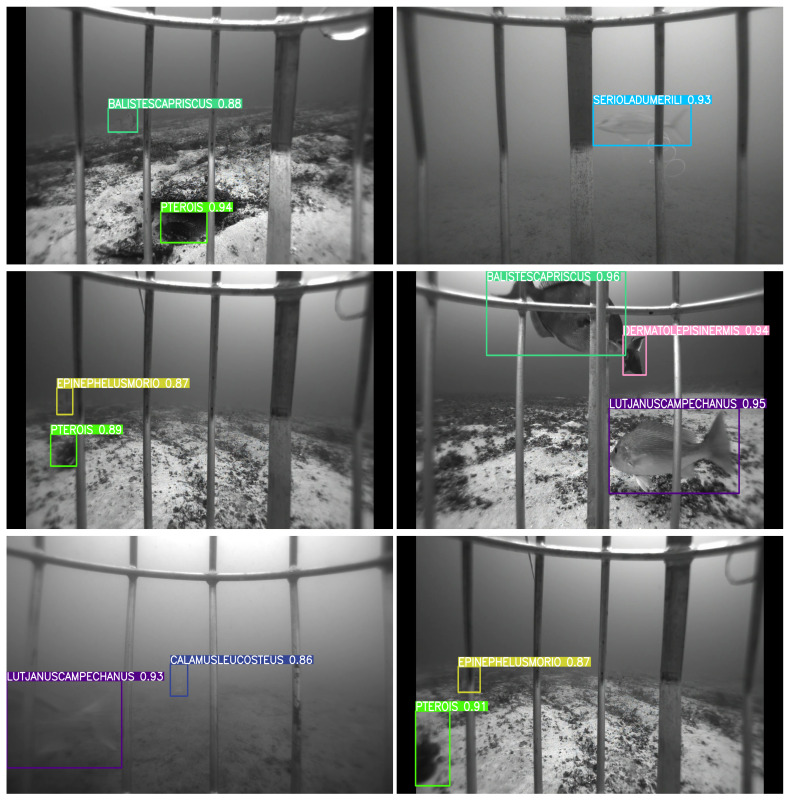
Detection images for YOLOv8-TF in SEAMAPD21. High confidence is exhibited in the detection of all fish species within each image.

Figure 7, Figure 8, Figure 9 and Figure 10 depict detection maps for fish species in underwater environments using SEAMAPD21. The images are generated using the established YOLOv8s, YOLOv8m, YOLOv8l, and the proposed YOLOv8-TF. The numbers after the names refer to the version of the YOLO model. Notably, YOLOv8-TF as shown in Figure 10 exhibits superior detection performance compared to other models, as shown by the higher mAP values in Table 1. In certain images, even humans may find it challenging to detect missed fish. For instance, the bottom two images in Figure 8 and the upper left corner of Figure 9 may not be identifiable or verifiable by a human.

Table 4 presents an ablation study conducted on the proposed YOLOv8-TF technique. The removal of the transformer block, depicted in the backbone and neck sections of Figure 1, results in reduced accuracy for YOLOv8-TF with class-aware (ca) loss and modified depth scale of backbone in C2f structure. Specifically, the mAP_0.5_ is 82.8%, and mAP_0.5–0.95_ is 54.8%. Furthermore, upon removing the class aware (ca) loss function from YOLOv8-TF, the accuracy of YOLOv8-TF with modified depth scale of backbone in C2f structure, without ca loss and trans block decreases to an mAP_0.5_ of 81.8% and mAP_0.5–0.95_ of 53.2%. Furthermore, parameters in million (params) and FPS (frames per second) are reported for ablation analysis. It can be observed that proposed YOLOV8-TF shows higher mean average precision compared to other versions consuming more parameters of 30.56 M and slower FPS of 116. It is because of involvement of transformer block in backbone and neck, using class aware loss function along with wise iou v3 loss, and implementing increased depth scale in the backbone of proposed YOLOv8-TF.

## 5. Discussion

For proposed YOLOv8-TF on SEAMAPD21, we have listed estimation of parameter count, GFLOPS, and FPS in Table 1. Similarly, Table 4 shows estimation of params (parameters in millions) and FPS (frames per second) for ablation analysis on YOLOv8-TF. It can be observed that proposed YOLOv8-TF outperforms other compared methods in terms of mean average precision (mAP). However, it suffers from increased parameter counts and slower FPS. Due to attention mechanism involved in transformer, addition of increased depth scale, involvement of class imbalance loss along with wise iou v3 loss, proposed YOLOv8-TF is influenced by increased number of parameters. An increase in the number of parameters leads to slower recognition speed because more resources are needed for each inference. Our model shows a higher accuracy for increase in the number of parameter counts and a decrease in the FPS, which result in an higher inference time. This model requires substantial memory because of higher number of parameters compared to other baseline models, such asYOLOV8m, YOLOv8s, and YOLOv8n. It may limit its use in resource-constrained environments. Our model may need optimization to address issues of slower inference time and higher memory usage in resource-constrained environments like embedded systems.

## 6. Conclusions

This study presents an enhanced approach, YOLOv8-TF, for fish species recognition in underwater environments. The effectiveness of the proposed YOLOv8-TF is evaluated through experiments conducted on the Southeast Area Monitoring and Assessment Program Dataset 2021 (SEAMAPD21), which comprises an underwater fish species dataset obtained from the Gulf of Mexico. The experimental results highlight the superiority of YOLOv8-TF compared to existing YOLOv8-based techniques. The method’s effectiveness is improved by adjusting the backbone’s depth scale, and adding a transformer block to both the backbone and neck sections further enhances the recognition of different fish species. Moreover, class-aware (ca) loss further enhances the detection performance by helping mitigate the class imbalance issue in the SEAMAPD21 dataset. We compare different YOLO models to illustrate the performance improvements achieved by YOLOv8-TF.

Future research directions will concentrate on building a lightweight model that can be deployed in resource-constrained environments and devices, aiming to enhance real-time processing capabilities and achieve higher accuracy. Furthermore, we plan to significantly ramp up the complexity and scope of our training data by incorporating a full training library that includes all available video annotations. This expansion will allow YOLOv8-TF to leverage temporal information inherent in video data and enrich the model’s understanding of fish behavior and movement patterns, significantly enhancing our model’s capabilities in fish tracking and counting across multiple images or videos. Such advancements will propel our ability to monitor and understand aquatic ecosystems to new heights and open the way for groundbreaking marine biology and conservation applications.

## Figures and Tables

**Table 1:** The mean average precision (mAP%) was computed for 121 species using the SEAMAPD21 dataset (see Table A1).

Method	mAP_0.5_	mAP_0.5:0.95_	Parameters	GFLOPS	FPS
MobileNetv3-large	-	32.51	-	-	105
VGG300	-	48.99	-	-	67
VGG512	-	52.75	-	-	54
YOLOv5s	71.9	43.9	7.37 M	17.1	117
YOLOv5m	75.6	47.9	21.37 M	49.5	110
YOLOv5l	78.5	50.6	46.80 M	109.9	103
YOLOv5enh	81.1	53.0	61.30 M	151.0	99
YOLOv8n	72.1	45.4	3.66 M	11.2	146
YOLOv8s	76.1	49.6	11.21 M	29.1	137
YOLOv8m	80.3	52.7	25.91 M	79.1	128
YOLOv8l	81.1	53.4	43.70 M	151.0	120
YOLOv10l	84.2	58.5	25.92 M	127.4	125
YOLOv8-TF	87.9	61.2	30.56 M	195.7	116

**Table 2:** The mean average precision (mAP%) was computed for 20 classes using the pascal VOC dataset.

Method	mAP_0.5_	mAP_0.5:0.95_	Parameters	FPS
YOLOv8n	83.90	53.70	3.93 M	194
YOLOv8s	87.65	54.80	14.81 M	168
YOLOv8m	89.10	54.10	26.49 M	155
YOLOv8l	91.70	56.40	47.31 M	141
YOLOv10l	92.40	57.12	25.79 M	147
YOLOv8-TF	94.60	58.21	30.51 M	130

**Table 3:** The mean average precision (mAP%) was computed for 80 classes using the MSCOCO dataset.

Method	mAP_0.5_	mAP_0.5:0.95_	Parameters	FPS
YOLOv8n	47.70	32.40	4.07 M	242
YOLOv8s	59.44	42.43	14.83 M	216
YOLOv8m	62.90	45.70	26.86 M	190
YOLOv8l	67.20	49.60	47.36 M	153
YOLOv10l	68.20	50.50	25.88 M	162
YOLOv8-TF	69.50	51.80	30.54 M	139

**Table 4:** Ablation analysis.

YOLOv8-TF				mAP_0.5_	mAP_0.5:0.95_	Params	FPS
Depth Scale	CA Loss	Trans	Wise IoU v3 Loss				
✓	✕	✕	✕	81.8	53.2	26.87 M	126
✓	✓	✕	✕	82.8	54.8	27.12 M	123
✓	✓	✓	✕	86.2	59.2	30.52 M	118
✓	✓	✓	✓	87.9	61.2	30.56 M	116

## Data Availability

The dataset is available at https://github.com/SEFSC/SEAMAPD21 (accessed on 8 March 2025). The given link is updated every time. So, it may have more datasets than we have used for this experiment. In addition, the source of this work will be made publicly available at https://github.com/SEFSC/FATES-ATI-EnhancedYOLOv8 (accessed on 8 March 2025).

## Abbreviations

The following abbreviations are used in this manuscript:

YOLOv8	You Only Look Once, version 8
YOLOv8enh	enhanced form of You Only Look Once, version 8
SEAMAPD21	The Southeast Area Monitoring and Assessment Program Dataset 2021
trans	Transformer block

## Appendix A

**Table A1:** mAP (in %) for various methods on SEAMAPD21 dataset [4,5].

Species	MobileNetv3	VGG300	VGG512	YOLOv5m	YOLOv5l	YOLOv5enh	YOLOv8s	YOLOv8m	YOLOv8l	YOLOv10l	YOLOv8-TF
ACANTHURUSCOERULEUS	-	-	-	0.96	0.54	0.18	8.04	0	20.0	14.3	43.9
ACANTHURUS	20.5	54.55	59.32	48.8	49.8	51.4	47.5	48.3	48.1	56.3	65.3
ALECTISCILIARIS	72.73	36.36	29.9	64.4	64.7	62.7	62.2	70.4	65.4	68.2	65.9
ANISOTREMUSVIRGINICUS	-	-	-	15.9	15.7	33.8	6.7	23.7	39.7	49.6	66.3
ANOMURA	0	61.36	72.73	74.1	72.4	81.2	78.1	85.2	85.7	82.2	75.9
ANTHIINAE	-	-	-	38.2	49.0	44.9	38.2	43.6	49.7	55.5	53.0
ARCHOSARGUSPROBATOCEPHALUS	15.02	45.45	54.13	38.3	44.8	47.5	47.1	50.5	47.1	50.5	61.2
BALISTESCAPRISCUS	67.53	73.42	74.31	70.0	70.8	72.6	73.1	75.1	75.5	76.7	77.3
BALISTESVETULA	20.7	37.26	42.92	54.7	63.5	74.2	81.6	53.0	58.2	83.2	67.0
BODIANUSPULCHELLUS	38.07	55.63	59.21	61.4	61.4	62.0	61.4	65.4	66.4	66.4	71.9
BODIANUSRUFUS	0	20.41	41.53	37.7	35.3	39.4	43.3	36.7	37.0	47.8	57.4
CALAMUSBAJONADO	0	78.6	89.6	89.5	79.6	79.6	89.5	79.6	79.6	79.6	63.3
CALAMUSLEUCOSTEUS	54.86	69.24	73.16	71.8	72.2	72.7	75.7	77.1	77.5	77.2	80.1
CALAMUSNODOSUS	7.57	6.89	10.3	74.7	75.9	77.3	77.2	78.1	78.7	81.7	80.0
CALAMUSPRORIDENS	15.34	18.64	12.75	64.5	66.4	67.2	68.5	70.9	70.9	73.5	74.8
CALAMUS	26.73	48.4	58.22	58.6	64.1	62.8	59.3	63.8	64.8	67.6	62.1
CANTHIDERMISSUFFLAMEN	2.6	47.73	48.42	45.8	49.1	52.8	45.7	51.5	52.8	53.6	49.6
CANTHIGASTERROSTRATUS	-	-	-	45.0	53.7	65.0	32.5	51.2	47.4	66.9	57.7
CARANXBARTHOLOMAEI	46.62	50.64	54.35	58.1	60.5	62.6	59.7	58.6	60.7	64.6	76.1
CARANXCRYSOS	19.13	48.94	45.56	53.7	54.8	58.1	57.0	57.5	56.6	60.6	59.0
CARANXRUBER	0	84.85	54.55	68.5	75.0	73.9	74.9	77.0	71.5	76.1	74.0
CARCHARHINUSFALCIFORMIS	100	54.55	54.55	81.6	84.3	83.5	78.3	83.2	83.2	83.0	78.5
CARCHARHINUSPEREZI	100	100	100	-	-	-	-		-	-	
CARCHARHINUSPLUMBEUS	0	6.06	36.36	57.3	59.8	67.1	60.5	66.4	62.8	65.3	50.0
CAULOLATILUSCHRYSOPS	41.85	42.43	39.22	73.0	72.8	75.2	74.2	79.9	77.8	80.1	79.1
CAULOLATILUSCYANOPS	10.88	9.32	10.86	68.1	69.7	70.0	70.5	71.3	72.4	73.3	73.0
CENTROPRISTISOCYURA	-	-	-	63.9	65.5	67.5	68.8	75.4	74.3	74.1	74.0
CEPHALOPHOLISCRUENTATA	31.1	70.63	65.14	54.6	55.3	54.7	53.8	56.6	60.4	56.1	51.5
CHAETODONOCELLATUS	-	-	-	29.8	31.7	35.4	25.2	31.2	31.3	48.8	50.1
CHAETODONSEDENTARIUS	9.09	9.4	16.52	48.4	50.0	52.6	47.5	51.4	50.7	59.3	53.7
CHAETODON	-	-	-	13.4	19.6	32.3	0.57	19.0	30.6	43.2	40.7
CHROMISENCHRYSURUS	1.23	0	42.08	19.8	16.8	12.8	24.1	9.95	17.0	27.6	51.3
CHROMISINSOLATUS	100	100	11.76	48.3	65.0	44.4	46.0	40.0	57.3	35.5	58.5
CHROMIS	-	-	-	2.49	0	0	1.81	1.53	7.46	0.34	32.3
DERMATOLEPISINERMIS	17.44	6.43	13.01	78.7	80.6	81.0	83.6	85.1	8.44	87.2	87.0
DIODONTIDAE	100	100	100	3.73	19.9	9.95	14.9	9.95	9.95	59.7	75.8
DIPLECTRUMFORMOSUM	27.72	44.46	60.04	44.6	47.6	49.2	45.1	47.6	50.2	56.4	58.6
EPINEPHELUSADSCENSIONIS	21.44	63.68	75.8	41.1	45.8	46.6	42.1	45.5	50.5	58.5	64.5
EPINEPHELUSFLAVOLIMBATUS	90.85	90.76	90.41	82.4	82.1	85.3	87.1	89.1	88.6	86.5	89.1
EPINEPHELUSMORIO	33.69	37.5	35.33	73.5	74.5	77.1	77.1	78.3	79.3	81.6	80.2
EPINEPHELUSNIGRITUS	100	100	100	64.0	61.8	56.8	67.1	62.0	65.5	64.5	69.8
EPINEPHELUS	36.36	63.64	63.64	46.7	48.0	55.4	62.0	57.9	63.0	66.5	69.2
EQUETUSLANCEOLATUS	100	100	100	-	-		-	-		-	69.2
EQUETUSUMBROSUS	39.5	73.26	73.64	66.3	-	71.1	71.5	71.1	73.3	74.9	70.8
GONIOPLECTRUSHISPANUS	81.82	85.71	84.03	0	0	0	0	0	0	0	80.6
GYMNOTHORAXMORINGA	-	-	-	48.3	55.2	56.0	52.4	57.0	54.4	60.9	66.3
GYMNOTHORAXSAXICOLA	-	-	-	62.2	61.1	63.7	59.6	66.1	66.2	68.4	70.2
HAEMULONAUROLINEATUM	55.57	68.3	75.68	62.6	62.9	67.0	63.9	68.4	69.2	68.8	67.9
HAEMULONFLAVOLINEATUM	0	24.11	67.85	34.1	35.4	39.6	44.1	37.2	49.4	57.6	51.6
HAEMULONMACROSTOMUM	50	100	100	44.2	48.0	53.0	39.2	43.8	61.0	65.4	80.5
HAEMULONMELANURUM	53.03	77.29	84.94	64.2	66.8	68.1	64.6	69.1	72.4	71.9	70.0
HAEMULONPLUMIERI	39.48	47.68	64.31	42.3	41.9	50.7	44.0	54.0	50.1	57.7	65.8
HALICHOERESBATHYPHILUS	9.27	26.55	52.36	39.7	48.1	47.0	51.4	52.2	51.9	60.7	37.9
HALICHOERESBIVITTATUS	-	-	-	22.5	26.6	31.4	21.7	25.6	27.9	40.3	41.4
HALICHOERESGARNOTI	-	-	-	23.2	21.3	29.9	28.7	35.0	39.6	34.2	47.9
HALICHOERES	-	-	-	32.1	-	37.1	31.5	41.0	38.9	45.0	45.2
HOLACANTHUSBERMUDENSIS	11.02	18.65	23.38	92.8	-	68.9	68.3	70.0	70.7	74.1	75.1
HOLACANTHUS	-	-	-	31.5	33.9	39.2	34.0	44.6	44.6	36.8	70.9
HOLANTHIUSMARTINICENSIS	-	-	-	78.4	83.5	47.5	32.7	41.8	45.3	58.6	54.9
HOLOCENTRUS	0	84.22	93.78	48.3		62.5	50.9	61.9	60.0	64.1	54.0
HYPOPLECTRUSGEMMA	-	-	-	13.5	19.9	33.4	13.1	20.9	27.3	43.0	20.0
HYPOPLECTRUS	-	-	-	25.3	29.6	34.2	13.1	29.9	22.4	51.2	61.6
HYPOPLECTRUSUNICOLOR	0	6.06	33.64	53.2	57.0	67.7	62.9	69.0	67.0	68.6	56.2
IOGLOSSUS	-	-	-	37.7	43.3	47.2	29.0	39.3	41.2	56.8	55.8
KYPHOSUS	24.76	18.58	27.91	47.5	45.7	63.3	59.5	58.6	53.3	62.2	27.6
LACHNOLAIMUSMAXIMUS	9.8	12.73	9.32	59.1	60.3	60.1	63.2	60.0	63.6	57.0	62.4
LACTOPHRYSTRIGONUS	-	-	-	30.7	35.1	34.8	26.3	32.8	40.5	51.5	72.3
LIOPROPOMAEUKRINES	3.03	20.78	21.56	70.8	65.0	69.3	65.0	76.3	70.5	71.3	80.8
LUTJANUSANALIS	41.12	28.02	21.21	57.3	63.5	58.1	57.6	61.7	59.2	59.9	69.3
LUTJANUSAPODUS	0	44.44	54.55	48.9	40.3	41.9	30.4	29.4	47.2	54.1	30.2
LUTJANUSBUCCANELA	62.02	77.09	81.72	70.9	71.7	73.7	74.8	76.9	76.5	79.0	77.1
LUTJANUSCAMPECHANUS	37.58	49.26	50.18	68.9	69.7	72.3	71.5	74.2	74.3	75.5	75.5
LUTJANUSGRISEUS	16.3	40.1	54.56	58.0	60.1	61.7	60.4	61.8	63.2	67.7	67.2
LUTJANUSSYNAGRIS	11.81	13.16	9.49	62.3	63.0	65.4	62.6	64.0	65.2	71.2	72.4
LUTJANUS	-	-	-	16.9	28.7	38.1	34.8	41.8	37.4	27.4	43.1
LUTJANUSVIVANUS	70.66	36.56	45.45	87.5	86.6	87.5	85.9	90.1	92.9	91.1	79.3
MALACANTHUSPLUMIERI	-	-	-	56.1	56.8	59.1	55.2	59.4	60.1	63.5	70.1
MULLOIDICHTHYSMARTINICUS	0	72.73	72.73	54.3	85.2	85.2	73.1	84.2	75.3	70.0	49.7
MURAENARETIFERA	70.94	89.05	71.73	63.7	56.6	69.5	63.1	66.0	71.7	76.1	69.0
MYCTEROPERCABONACI	45.45	90.91	90.91	48.0	47.1	52.3	44.4	56.1	52.9	59.2	54.8
MYCTEROPERCAINTERSTIALIS	0	70.91	39.09	75.6	78.3	77.7	76.7	73.2	74.3	80.1	82.3
MYCTEROPERCAINTERSTITIALIS	68.82	63.42	69.87	67.0	70.8	69.3	68 .5	73.2	72.6	71.9	76.1
MYCTEROPERCAMICROLEPIS	-	-	-	59.7	49.7	59.7	69.7	69.7	69.7	69.7	81.0
MYCTEROPERCAPHENAX	26.26	40.52	41.48	68.1	69.5	70.1	70.3	72.3	72.5	74.6	77.6
MYCTEROPERCA	14.55	54.65	45.86	31.9	31.9	35.9	32.2	42.4	51.0	37.6	30.4
OCYURUSCHRYSURUS	21.24	42.16	55.55	39.6	43.9	46.5	41.5	46.0	45.6	52.1	51.2
OPHICHTHUSPUNCTICEPS	-	-	-	57.9	60.5	61.9	60.3	63.3	67.5	65.3	71.9
OPISTOGNATHUSAURIFRONS	-	-	-	24.8	31.6	38.7	18.3	30.3	30.6	41.7	44.6
PAGRUSPAGRUS	21.21	28.55	31.68	64.7	65.6	67.8	66.7	69.5	69.7	72.6	71.6
PARANTHIASFURCIFER	54.55	56.21	15.36	34.1	44.7	37.2	33.6	40.3	25.1	41.5	43.1
POMACANTHUSARCUATUS	18.92	17.43	18.81	64.0	64.4	66.9	67.4	69.2	67.7	73.4	68.2
POMACANTHUSPARU	27.86	59.82	71.83	68.9	66.5	74.6	73.6	73.6	72.2	74.9	71.9
POMACANTHUS	0	0	63.64	0	0	0	0	0	0	0	28.9
POMACENTRIDAE	0	10.99	25.77	30.5	32.6	37.3	27.0	32.3	33.8	42.2	44.6
POMACENTRUSPARTITUS	-	-	-	11.0	19.9	24.2	10.1	12.8	18.8	28.6	33.9
POMACENTRUS	-	-	-	0	1.34	1.56	0	0	0	27.5	0
PRIACANTHUSARENATUS	-	-	-	52.2	54.7	24.2	42.4	39.8	55.2	60.4	82.6
PRISTIGENYSALTA	0	12.33	12.86	64.6	67.2	66.9	65.1	67.8	68.7	75.1	76.3
PRISTIPOMOIDESAQUILONARIS	3.58	15.44	18.17	85.7	50.2	51.1	48.3	50.8	52.4	52.4	51.3
PSEUDUPENEUSMACULATUS	-	-	-	64.6	69.6	74.6	50.0	70.5	40.6	67.5	40.5
PTEROIS	32.83	23.57	22.9	75.9	78.2	78.7	81.6	83.3	85.1	83.4	82.9
RACHYCENTRONCANADUM	45.45	0	27.72	89.5	83.0	73.1	79.8	82.3	80.2	88.4	74.9
RHOMBOPLITESAURORUBENS	44.73	60.6	67.29	59.0	60.3	61.8	62.1	63.8	64.5	64.6	64.0
RYPTICUSMACULATUS	50.79	70.94	73.96	61.1	63.2	65.1	63.8	66.6	69.1	67.5	69.3
SCARIDAE	-	-	-	1.55	0	1.14	9.97	0.27	1.21	0.21	13.5
SCARUSVETULA	-	-	-	40.0	34.8	34.9	40.6	38.3	31.0	56.0	52.0
SERIOLADUMERILI	51.62	61.19	59.48	69.0	69.6	70.6	72.3	73.5	74.1	74.3	73.0
SERIOLAFASCIATA	45.62	61.89	54.66	61.8	63.4	65.2	64.9	69.3	68.3	68.2	69.6
SERIOLARIVOLIANA	51.55	60.93	66.46	69.0	71.8	72.7	72.1	74.6	74.5	76.0	74.1
SERIOLA	-	-	-	23.2	22.6	39.8	32.2	38.3	17.6	43.6	43.3
SERIOLAZONATA	-	-	-	69.7	89.5	69.7	69.7	79.6	69.7	79.6	57.8
SERRANUSANNULARIS	-	-	-	64.1	65.1	67.9	65.0	70.2	70.1	77.8	79.0
SERRANUSPHOEBE	-	-	-	40.6	40.7	44.6	41.5	40.7	45.0	47.5	54.9
SPARIDAE	-	-	-	19.8	54.7	59.7	54.8	43.1	42.3	57.2	69.7
SPARISOMAAUROFRENATUM	-	-	-	0.72	0.72	0	0.83	0	1.03	8.6	0.60
SPARISOMAVIRIDE	-	-	-	30.1	38.0	38.7	38.6	46.5	39.4	47.6	0
SPHYRAENABARRACUDA	0	45.45	45.45	71.1	73.4	78.4	72.1	71.8	83.7	84.1	74.4
STENOTOMUSCAPRINUS	-	-	-	0.88	0	1.08	0.4	13.1	2.83	17.3	11.5
SYACIUM	-	-	-	79.1	79.1	76.5	79.5	81.2	8.44	88.4	86.7
SYNODONTIDAE	-	-	-	50.6	58.0	63.4	38.7	59.3	5.57	68.4	76.8
THALASSOMABIFASCIATUM	-	-	-	3.36	6.13	11.4	2.21	9.9	6.58	34.2	38.2
UPENEUSPARVUS	1.07	17.65	37.36	25.6	28.9	26.3	26.5	33.4	22.6	31.0	53.1
XANTHICHTHYSRINGENS	23.21	100	100	78.3	81.0	83.0	77.7	84.7	8.73	87.5	74.7

## References

[1] Changa C.M., rong Fanga W., Jaob R.C., Shyuc C.Z., Liaoc I.C. (2004). Development of an intelligent feeding controller for indoor intensive culturing of eel. Aquac. Eng..

[2] Cabreira A.G., Tripode M., Madirolas A. (2009). Artificial neural networks for fish-species identification. ICES J. Mar. Sci..

[3] Alaba S., Shah C., Nabi M., Ball J., Moorhead R., Han D., Prior J., Campbell M., Wallace F. (2023). Semi-supervised learning for fish species recognition. Proceedings of the Ocean Sensing and Monitoring XV.

[4] Alaba S.Y., Nabi M., Shah C., Prior J., Campbell M.D., Wallace F., Ball J.E., Moorhead R. (2022). Class-aware fish species recognition using deep learning for an imbalanced dataset. Sensors.

[5] Shah C., Alaba S.Y., Nabi M.M., Prior J., Campbell M., Wallace F., Ball J.E., Moorhead R., Hou W., Mullen L.J. (2023). An enhanced YOLOv5 model for fish species recognition from underwater environments. Proceedings of the Ocean Sensing and Monitoring XV.

[6] Shah C., Alaba S.Y., Nabi M.M., Caillouet R., Prior J., Campbell M., Wallace F., Ball J.E., Moorhead R., Hou W., Mullen L.J. (2023). MI-AFR: Multiple instance active learning-based approach for fish species recognition in underwater environments. Proceedings of the Ocean Sensing and Monitoring XV.

[7] Prior J., Campbell M., Dawkins M., Mickle P., Moorhead R., Alaba S., Shah C., Salisbury J., Rademacher K., Felts A. (2023). Estimating precision and accuracy of automated video post-processing: A step towards implementation of AI/ML for optics-based fish sampling. Front. Mar. Sci..

[8] Jalali M.A., Ierodiaconou D., Monk J., Gorfine H., Rattray A. (2015). Predictive mapping of abalone fishing grounds using remotely-sensed LiDAR and commercial catch data. Fish. Res..

[9] Churnside J.H., Wells R., Boswell K.M., Quinlan J.A., Marchbanks R.D., McCarty B.J., Sutton T.T. (2017). Surveying the distribution and abundance of flying fishes and other epipelagics in the northern Gulf of Mexico using airborne lidar. Bull. Mar. Sci..

[10] Boswell K.M., Wilson M.P., Cowan J.H. (2008). A semiautomated approach to estimating fish size, abundance, and behavior from dual-frequency identification sonar (DIDSON) data. N. Am. J. Fish. Manag..

[11] Villon S., Chaumont M., Subsol G., Villéger S., Claverie T., Mouillot D. (2016). Coral reef fish detection and recognition in underwater videos by supervised machine learning: Comparison between Deep Learning and HOG+ SVM methods. Proceedings of the International Conference on Advanced Concepts for Intelligent Vision Systems.

[12] Bicknell A.W., Godley B.J., Sheehan E.V., Votier S.C., Witt M.J. (2016). Camera technology for monitoring marine biodiversity and human impact. Front. Ecol. Environ..

[13] Shortis M., Harvey E., Abdo D. (2016). A review of underwater stereo-image measurement for marine biology and ecology applications. Oceanography and Marine Biology.

[14] Panetta K., Kezebou L., Oludare V., Agaian S. (2022). Comprehensive Underwater Object Tracking Benchmark Dataset and Underwater Image Enhancement With GAN. IEEE J. Ocean. Eng..

[15] Slonimer A.L., Dosso S.E., Albu A.B., Cote M., Marques T.P., Rezvanifar A., Ersahin K., Mudge T., Gauthier S. (2023). Classification of Herring, Salmon, and Bubbles in Multifrequency Echograms Using U-Net Neural Networks. IEEE J. Ocean. Eng..

[16] Ntouskos V., Mertikas P., Mallios A., Karantzalos K. (2023). Seabed Classification From Multispectral Multibeam Data. IEEE J. Ocean. Eng..

[17] Xiao F., Yuan F., Huang Y., Cheng E. (2022). Turbid underwater image enhancement based on parameter-tuned stochastic resonance. IEEE J. Ocean. Eng..

[18] Gu K., Liu J., Shi S., Xie S., Shi T., Qiao J. (2022). Self-organizing multichannel deep learning system for river turbidity monitoring. IEEE Trans. Instrum. Meas..

[19] Zeng L., Sun B., Zhu D. (2021). Underwater target detection based on Faster R-CNN and adversarial occlusion network. Eng. Appl. Artif. Intell..

[20] Harden Jones F. (1963). The reaction of fish to moving backgrounds. J. Exp. Biol..

[21] (2022). SWIPENET: Object detection in noisy underwater scenes. Pattern Recognit..

[22] Cardaillac A., Ludvigsen M. (2023). Camera-sonar combination for improved underwater localization and mapping. IEEE Access.

[23] Almanza-Medina J.E., Henson B., Zakharov Y.V. (2021). Deep learning architectures for navigation using forward looking sonar images. IEEE Access.

[24] Chang C.C., Ubina N.A., Cheng S.C., Lan H.Y., Chen K.C., Huang C.C. (2022). A Two-Mode Underwater Smart Sensor Object for Precision Aquaculture Based on AIoT Technology. Sensors.

[25] Wang Y., Yu X., An D., Wei Y. (2021). Underwater image enhancement and marine snow removal for fishery based on integrated dual-channel neural network. Comput. Electron. Agric..

[26] Ju Y., Xiao J., Zhang C., Xie H., Luo A., Zhou H., Dong J., Kot A.C. (2025). Towards marine snow removal with fusing Fourier information. Inf. Fusion.

[27] Kaneko R., Sato Y., Ueda T., Higashi H., Tanaka Y. Marine Snow Removal Benchmarking Dataset. Proceedings of the 2023 Asia Pacific Signal and Information Processing Association Annual Summit and Conference (APSIPA ASC).

[28] Debnath B., Ebu I.A., Biswas S., Gurbuz A.C., Ball J.E. Fmcw radar range profile and micro-doppler signature fusion for improved traffic signaling motion classification. Proceedings of the 2024 IEEE Radar Conference (RadarConf24).

[29] Xu W., Matzner S. Underwater fish detection using deep learning for water power applications. Proceedings of the 2018 International Conference on Computational Science and Computational Intelligence (CSCI).

[30] Nabi M., Shah C., Alaba S.Y., Prior J., Campbell M.D., Wallace F., Moorhead R., Ball J.E. Probabilistic model-based active learning with attention mechanism for fish species recognition. Proceedings of the OCEANS 2023-MTS/IEEE US Gulf Coast.

[31] Shah C., Nabi M., Alaba S.Y., Prior J., Caillouet R., Campbell M.D., Wallace F., Ball J.E., Moorhead R. A zero shot detection based approach for fish species recognition in underwater environments. Proceedings of the OCEANS 2023-MTS/IEEE US Gulf Coast.

[32] Jäger J., Rodner E., Denzler J., Wolff V., Fricke-Neuderth K. SeaCLEF 2016: Object Proposal Classification for Fish Detection in Underwater Videos. Proceedings of the CLEF (Working Notes).

[33] Carion N., Massa F., Synnaeve G., Usunier N., Kirillov A., Zagoruyko S., Vedaldi A., Bischof H., Brox T., Frahm J.M. (2020). End-to-End Object Detection with Transformers. Proceedings of the Computer Vision—ECCV 2020.

[34] Fang Y., Liao B., Wang X., Fang J., Qi J., Wu R., Niu J., Liu W. (2021). You only look at one sequence: Rethinking transformer in vision through object detection. Adv. Neural Inf. Process. Syst..

[35] Wang C.Y., Bochkovskiy A., Liao H.Y.M. Scaled-YOLOv4: Scaling Cross Stage Partial Network. Proceedings of the IEEE/CVF Conference on Computer Vision and Pattern Recognition (CVPR).

[36] Redmon J., Divvala S., Girshick R., Farhadi A. You only look once: Unified, real-time object detection. Proceedings of the IEEE Conference on Computer Vision and Pattern Recognition.

[37] Feng J., Jin T. (2024). CEH-YOLO: A composite enhanced YOLO-based model for underwater object detection. Ecol. Inform..

[38] Li Y., Li Q., Pan J., Zhou Y., Zhu H., Wei H., Liu C. (2024). SOD-YOLO: Small-Object-Detection Algorithm Based on Improved YOLOv8 for UAV Images. Remote Sens..

[39] Liu W., Anguelov D., Erhan D., Szegedy C., Reed S., Fu C.Y., Berg A.C. (2016). Ssd: Single shot multibox detector. Proceedings of the Computer Vision—ECCV 2016: 14th European Conference.

[40] Girshick R. Fast r-cnn. Proceedings of the IEEE International Conference on Computer Vision.

[41] Ortenzi L., Aguzzi J., Costa C., Marini S., D’Agostino D., Thomsen L., De Leo F.C., Correa P.V., Chatzievangelou D. (2024). Automated species classification and counting by deep-sea mobile crawler platforms using YOLO. Ecol. Inform..

[42] Jocher G., Stoken A., Chaurasia A., Borovec J., Kwon Y., Michael K., Changyu L., Fang J., Skalski P., Hogan A. (2021). ultralytics/yolov5: V6. 0-YOLOv5n’Nano’models, Roboflow integration, TensorFlow export, OpenCV DNN support. Zenodo.

[43] Lin T.Y., Maire M., Belongie S., Hays J., Perona P., Ramanan D., Dollár P., Zitnick C.L. (2014). Microsoft coco: Common objects in context. Proceedings of the Computer Vision—ECCV 2014: 13th European Conference.

[44] Jung H.K., Choi G.S. (2022). Improved YOLOv5: Efficient Object Detection Using Drone Images under Various Conditions. Appl. Sci..

[45] Wang H., Hu Z., Mo H., Zhao X. (2025). Enhanced nighttime nail detection using improved YOLOv5 for precision road safety. Sci. Rep..

[46] Varghese R., Sambath M. YOLOv8: A Novel Object Detection Algorithm with Enhanced Performance and Robustness. Proceedings of the 2024 International Conference on Advances in Data Engineering and Intelligent Computing Systems (ADICS).

[47] Zhang Y., Wu Z., Wang X., Fu W., Ma J., Wang G. Improved YOLOv8 Insulator Fault Detection Algorithm Based on BiFormer. Proceedings of the 2023 IEEE 5th International Conference on Power, Intelligent Computing and Systems (ICPICS).

[48] Li Y., Fan Q., Huang H., Han Z., Gu Q. (2023). A Modified YOLOv8 Detection Network for UAV Aerial Image Recognition. Drones.

[49] Ansah P.A.K., Appati J.K., Owusu E., Boahen E.K., Boakye-Sekyerehene P., Dwumfour A. (2025). SB-YOLO-V8: A Multilayered Deep Learning Approach for Real-Time Human Detection. Eng. Rep..

[50] Bi J., Li K., Zheng X., Zhang G., Lei T. (2025). SPDC-YOLO: An Efficient Small Target Detection Network Based on Improved YOLOv8 for Drone Aerial Image. Remote Sens..

[51] Yang X., Zhang S., Liu J., Gao Q., Dong S., Zhou C. (2021). Deep learning for smart fish farming: Applications, opportunities and challenges. Rev. Aquac..

[52] Villon S., Mouillot D., Chaumont M., Darling E.S., Subsol G., Claverie T., Villéger S. (2018). A deep learning method for accurate and fast identification of coral reef fishes in underwater images. Ecol. Inform..

[53] Rathi D., Jain S., Indu S. Underwater fish species classification using convolutional neural network and deep learning. Proceedings of the 2017 Ninth International Conference on Advances in Pattern Recognition (ICAPR).

[54] Shreesha S., Pai M.M., Pai R.M., Verma U. (2023). Pattern detection and prediction using deep learning for intelligent decision support to identify fish behaviour in aquaculture. Ecol. Inform..

[55] Zhou C., Xu D., Chen L., Zhang S., Sun C., Yang X., Wang Y. (2019). Evaluation of fish feeding intensity in aquaculture using a convolutional neural network and machine vision. Aquaculture.

[56] Abinaya N., Susan D., Sidharthan R.K. (2022). Deep learning-based segmental analysis of fish for biomass estimation in an occulted environment. Comput. Electron. Agric..

[57] Zambrano A.F., Giraldo L.F., Quimbayo J., Medina B., Castillo E. (2021). Machine learning for manually-measured water quality prediction in fish farming. PLoS ONE.

[58] Boulais O., Alaba S.Y., Ball J.E., Campbell M., Iftekhar A.T., Moorehead R., Primrose J., Prior J., Wallace F., Yu H. SEAMAPD21: A large-scale reef fish dataset for fine-grained categorization. Proceedings of the Eight Workshop on Fine-Grained Visual Categorization.

[59] Howard A., Sandler M., Chu G., Chen L.C., Chen B., Tan M., Wang W., Zhu Y., Pang R., Vasudevan V. Searching for mobilenetv3. Proceedings of the IEEE/CVF International Conference on Computer Vision.

[60] Simonyan K., Zisserman A. (2014). Very deep convolutional networks for large-scale image recognition. arXiv.

[61] Wang X., Xue G., Huang S., Liu Y. (2023). Underwater Object Detection Algorithm Based on Adding Channel and Spatial Fusion Attention Mechanism. J. Mar. Sci. Eng..

[62] Pachaiyappan P., Chidambaram G., Jahid A., Alsharif M.H. (2024). Enhancing Underwater Object Detection and Classification Using Advanced Imaging Techniques: A Novel Approach with Diffusion Models. Sustainability.

[63] Sung M., Yu S.C., Girdhar Y. Vision based real-time fish detection using convolutional neural network. Proceedings of the OCEANS 2017-Aberdeen.

[64] Jalal A., Salman A., Mian A., Shortis M., Shafait F. (2020). Fish detection and species classification in underwater environments using deep learning with temporal information. Ecol. Inform..

[65] (2023). Ultralytics. Ultralytics/ultralytics: New000YOLOv8 in PyTorch > ONNX > CoreML > TFLite.

[66] Lin T.Y., Dollár P., Girshick R., He K., Hariharan B., Belongie S. Feature Pyramid Networks for Object Detection. Proceedings of the 2017 IEEE Conference on Computer Vision and Pattern Recognition (CVPR).

[67] Liu S., Qi L., Qin H., Shi J., Jia J. Path Aggregation Network for Instance Segmentation. Proceedings of the 2018 IEEE/CVF Conference on Computer Vision and Pattern Recognition, Salt Lake City.

[68] Terven J., Córdova-Esparza D.M., Romero-González J.A. (2023). A Comprehensive Review of YOLO Architectures in Computer Vision: From YOLOv1 to YOLOv8 and YOLO-NAS. Mach. Learn. Knowl. Extr..

[69] Roy S.K., Sukul A., Jamali A., Haut J.M., Ghamisi P. (2024). Cross hyperspectral and LiDAR attention transformer: An extended self-attention for land use and land cover classification. IEEE Trans. Geosci. Remote Sens..

[70] Zhang Z., Lu X., Cao G., Yang Y., Jiao L., Liu F. ViT-YOLO:Transformer-Based YOLO for Object Detection. Proceedings of the 2021 IEEE/CVF International Conference on Computer Vision Workshops (ICCVW).

[71] Wu T., Dong Y. (2023). YOLO-SE: Improved YOLOv8 for Remote Sensing Object Detection and Recognition. Appl. Sci..

[72] Tong Z., Chen Y., Xu Z., Yu R. (2023). Wise-IoU: Bounding box regression loss with dynamic focusing mechanism. arXiv.

